# Radiomic Based Machine Learning Performance for a Three Class Problem in Neuro-Oncology: Time to Test the Waters?

**DOI:** 10.3390/cancers13112568

**Published:** 2021-05-24

**Authors:** Sarv Priya, Yanan Liu, Caitlin Ward, Nam H. Le, Neetu Soni, Ravishankar Pillenahalli Maheshwarappa, Varun Monga, Honghai Zhang, Milan Sonka, Girish Bathla

**Affiliations:** 1 Department of Radiology, University of Iowa Hospitals and Clinics, Iowa City, IA 52242, USA; neetu-soni@uiowa.edu (N.S.); ravishankar-pm@uiowa.edu (R.P.M.); girish-bathla@uiowa.edu (G.B.); 2 College of Engineering, University of Iowa, Iowa City, IA 52242, USA; yanan-liu@uiowa.edu (Y.L.); nam-h-le@uiowa.edu (N.H.L.); honghai-zhang@uiowa.edu (H.Z.); milan-sonka@uiowa.edu (M.S.); 3 Department of Biostatistics, University of Iowa, Iowa City, IA 52242, USA; caitlin-e-ward@uiowa.edu; 4 Department of Medicine, University of Iowa Hospitals and Clinics, Iowa City, IA 52242, USA; varun-monga@uiowa.edu

**Keywords:** radiomics, MRI, glioblastoma, metastases, CNS lymphoma, machine learning, texture

## Abstract

**Simple Summary:**

Prior radiomic studies have addressed a two-class tumor classification problem (glioblastoma (GBM) versus primary CNS lymphoma (PCNSL) or GBM versus metastasis). However, this approach is prone to bias and excludes other common brain tumor types. We addressed a real-life clinical problem by including the three most common brain tumor types (GBM, PCNSL, and metastasis). We investigated two key issues using different MRI sequence combinations: performance variation based on tumor subregions (necrotic, enhancing, edema and combined enhancing, and necrotic masks), and performance metrics based on the chosen classifier model/feature selection combination. Our study provides evidence that radiomics-based three-class tumor differentiation is feasible, and that embedded models perform better than those with a priori feature selection. We found that T1 contrast enhanced is the single best sequence with comparable performance to that of multiparametric MRI, and model performance varies based on tumor subregion and the combination of model/feature selection methods.

**Abstract:**

Prior radiomics studies have focused on two-class brain tumor classification, which limits generalizability. The performance of radiomics in differentiating the three most common malignant brain tumors (glioblastoma (GBM), primary central nervous system lymphoma (PCNSL), and metastatic disease) is assessed; factors affecting the model performance and usefulness of a single sequence versus multiparametric MRI (MP-MRI) remain largely unaddressed. This retrospective study included 253 patients (120 metastatic (lung and brain), 40 PCNSL, and 93 GBM). Radiomic features were extracted for whole a tumor mask (enhancing plus necrotic) and an edema mask (first pipeline), as well as for separate enhancing and necrotic and edema masks (second pipeline). Model performance was evaluated using MP-MRI, individual sequences, and the T1 contrast enhanced (T1-CE) sequence without the edema mask across 45 model/feature selection combinations. The second pipeline showed significantly high performance across all combinations (Brier score: 0.311–0.325). GBRM fit using the full feature set from the T1-CE sequence was the best model. The majority of the top models were built using a full feature set and inbuilt feature selection. No significant difference was seen between the top-performing models for MP-MRI (AUC 0.910) and T1-CE sequence with (AUC 0.908) and without edema masks (AUC 0.894). T1-CE is the single best sequence with comparable performance to that of multiparametric MRI (MP-MRI). Model performance varies based on tumor subregion and the combination of model/feature selection methods.

## 1. Introduction

Glioblastomas (GBM), primary central nervous system lymphomas (PCNSL), and parenchymal metastatic lesions account for the vast majority of malignant brain tumors in clinical neuro-oncology. Magnetic resonance imaging (MRI) is most commonly used for pre-operative characterization of these tumors [1,2]. However, the radiologically observed imaging features of these malignancies often overlap. Since the treatment strategies are different (resection followed by chemoradiation for GBM, chemotherapy for PCNSL, and chemotherapy/radiosurgery for metastatic lesions), early and accurate preoperative differentiation of these tumors is critical [2,3,4]. This is generally achieved through resection or brain biopsy. Brain biopsy is, however, not always optimal, with misdiagnosis and under-grading of tumors reported in 9.2 and 28% of neoplastic lesions, respectively [5]. The reported biopsy complication rate varies between 6 and 12%, with a mortality rate of 0–1.7% [6]. Expert human readers also have modest accuracy, which could be further improved with the available advanced imaging techniques and/or computational tools [4]. There is therefore a continued need for more accurate pre-operative diagnosis, which may be conducted non-invasively with more advanced imaging techniques or through artificial intelligence.

The use of radiomics in brain tumor classification could be extremely helpful for non-invasive diagnosis since it converts the sparse imaging data into big data (histogram, texture, and transformed features) using a voxel wise approach. Prior studies have explored the utility of MRI-derived radiomic features for brain tumor classification [7,8]. However, most of these studies have generally addressed a two-class problem, either GBM versus PCNSL [9,10,11] or GBM versus metastases [12], which is not a pragmatic approach since this presupposes accurate exclusion of one main category of tumor. The existence of overlapping texture features of a third pathology and its impact on model prediction and real-life performance therefore remain unaddressed. Even though such studies have shown good results, they do not reflect a real-life scenario and follow a more simplistic approach.

What also remain largely unknown are the impact of various machine learning techniques as well as the role of feature selection when dealing with large data in a three-class problem [11,12,13]. Similarly, the usefulness of separate segmentations of the enhancing and necrotic components with edema masks (a total of 3 masks) versus the whole tumor (necrotic plus enhancing) with edema masks (a total of 2 masks) and their impact on model performance in a three-class problem remain unexplored. The aim of our study was to address a three-class problem (GBM vs. PCNSL vs. metastases) using a radiomics-based approach on retrospective MP-MRI data. We additionally evaluated the impact of different feature selection and machine learning techniques on overall model accuracy. Finally, we addressed the relevance of different tumor masks for the same three-class problem.

## 2. Materials and Methods

### 2.1. Data Collection 

This was a retrospective study approved by the local institutional review board (IRB-ID 201912239). Between 2010–2018, consecutive patients above the age of 18 years were identified using a combination of electronic medical records and institutional cancer registries. Patients with pathologically confirmed GBM (WHO grade IV) and immunocompetent PCNSL were identified. Since lung and breast cancer account for most of the cases of brain metastases, the metastatic lesion cohort was confined to patients with a known lung or breast primary. Only these two metastatic tumor types were selected to reduce data heterogeneity as part of this pilot study in order to differentiate the three most common brain tumor types using radiomics. Eligibility criteria included preoperative MRI scans that all had multiparametric (axial T1W, T2W, FLAIR, ADC, and T1 contrast enhanced (CE)) sequences available; presence of a contrast enhancing tumor; and no prior history of treatment, biopsy, or surgical resection. Patients with non-enhancing tumors, tumors less than 1 cm in diameter, and motion artifact were excluded.

**Figure 1:**
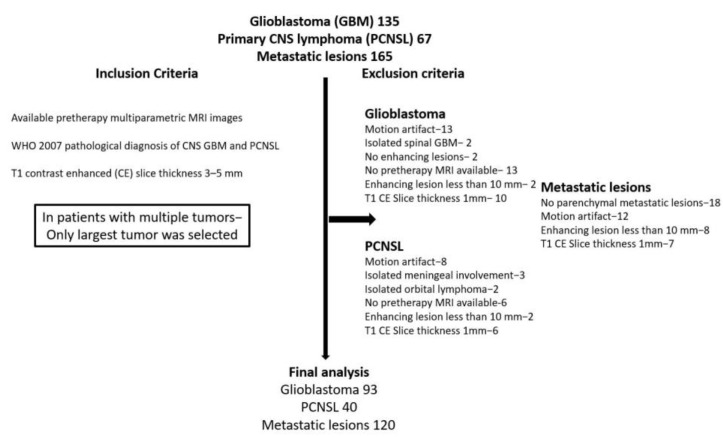
Patient selection criteria.

A total of 253 patients were included in the study (metastatic (*n* = 120, 47.4%), PCNSL (*n* = 40, 15.8%), and GBM (*n* = 93, 36.8%); Figure 1).

### 2.2. Image Acquisition 

Preoperative imaging was performed on 1.5T (232) and 3T (21) MRI system (Siemens, Erlangen, Germany). The acquisition protocol for brain tumor evaluation at our hospital includes pre-contrast axial T1W, T2W, FLAIR, diffusion weighted imaging with ADC maps, gradient echo, and tri-planar T1-CE images (details in Text S1). Five imaging sequences were evaluated in this study for the analysis: axial T1W, T2W, FLAIR, ADC map, and T1-CE.

### 2.3. Image Pre-Processing

Following image anonymization, DICOM images were converted to the NIfTI format. For enabling the volume of interest to be used with images from all MRI sequences, all images were resampled and aligned to the same spacing, resolution, and alignment using nearest neighbor resampling. Images were resampled to a 1 × 1 × 5 mm^3^ voxel size using the AFNI package (https://afni.nimh.nih.gov/ (accessed on 05/05/2021)) [14]. Due to large difference between slice thickness (5 mm) and in-plane spacing (0.5–0.75 mm) in our subjects, there was a risk of introducing artificial information and bias with upsampling and information loss with downsampling [15,16,17]. “As per image biomarker standardization initiative (IBSI) guidelines, in patients with large slice thickness compared to in plane voxel size dimensions, it may be beneficial to perform 2D interpolation. This is because if 3D interpolation is performed in these patients, there is a risk of information loss during downsampling (for example from 0.5 × 0.5 × 5 mm^3^ to 5 × 5 × 5 mm^3^). In addition, if upsampling is performed (for example from 0.5 × 0.5 × 5 mm^3^ to 0.5 × 0.5 × 0.5 mm^3^), there is a risk of introducing artificial information by inferencing a large number of voxels between slices.” [18]. As such, we performed standardized anisotropic resampling for all MRI sequences to ensure reproducibility as also performed in prior MRI radiomic studies [19,20]. Moreover, radiomic features have also been shown to be robust to different levels of pixel spacing and interpolation [21]. In addition, feature standardization (also performed in our study) has been shown to improve robustness of radiomic features beyond pixel spacing and interpolation [21]. All MRI image sequences were mutually registered to the pre-contrast T1W sequence using Advanced Normalization Tools (ANTs) (http://stnava.github.io/ANTs/ (accessed on 05/05/2021) [22] followed by min–max image intensity normalization to 0–255 using the feature scaling method available in the ANTs registration suite (http://stnava.github.io/ANTs/ (accessed on 05/05/2021). Min–max normalization is common method of intensity normalizations to preprocess data before model fitting within an intensity range of 0 and 255 (i.e., 256 different possible values) [23,24,25]. 

### 2.4. Tumor Segmentation/Region of Interest Delineation

Three-dimensional (3D) volumetric tumor segmentation was performed on axial T1-CE and FLAIR images by two radiologists (S.P. and G.B.) in consensus using an in-house developed semi-automatic tool, Layered Optimal Graph Image Segmentation for Multiple Objects and Surfaces (LOGISMOS) [26]. In patients with multiple lesions, only the largest lesion was segmented since this approach can provide reliable results by including regions containing a sufficient number of voxels, and the same approach has also been utilized in prior studies [27,28]. Four region of interests (masks) were created using T1-CE and FLAIR images: (i) whole tumor (enhancing plus necrotic); (ii) enhancing only; (iii) necrotic only; and (iv) peritumoral edema (details in the Supplementary Materials, Figure S1). These masks were superimposed on all five sequences (T1W, T2W, FLAIR, ADC map, and T1-CE).

### 2.5. Texture Feature Extraction

International Biomarker Standardization Initiative (IBSI) compliant radiomic features were extracted using Pyradiomics 3.0 [29]. As there were four masks and five imaging sequences, there were a total of 20 possible masks and sequence combinations. On each of these combinations, 107 radiomic features were extracted, consisting of 3D shape, first order, gray level co-occurrence matrix, gray level dependency matrix features, gray level run length matrix features, gray level size zone matrix features, and neighboring gray tone difference matrix features (details in Text S1). The analyzed 253 patient MR images yielded 1012 3D masks, for which radiomic features were obtained. About 4% of these masks referenced volumetrically small regions with less than four voxels in one of the x–y–z directions for which calculation of 3D texture features is of limited value when considered separately (43 masks—29 necrotic, 6 whole tumor, 6 enhancing, and 2 edema masks). In our case, to maintain feature-based consistency across subjects when used in the predictive models, the same set of 3D radiomic features was calculated for all available masks, including the 43 small ones (there were only 14 3D radiomic features out of a total of 107 features extracted). Details are provided in the Supplementary Materials (Table S1).

### 2.6. Feature Harmonization

As data were acquired from two types of MRI scanners (1.5 and 3T), there was the potential for the different signal intensities to lead to variations in the feature values. To account for this variation, the ComBat feature harmonization technique [30] was used prior to model fitting. This technique has been recently applied in radiomics studies and has been shown reduce feature differences between different scanners [31]. Feature harmonization was implemented using the neuroCombat package in R version 4.0.2, using the non-parametric adjustment method to avoid making any distributional assumptions about the features [32,33].

### 2.7. Feature Selection

Since large number of feature sets were extracted compared to the sample size, feature selection was performed to avoid collinearity and reduce dimension. These feature selection methods included: a linear combination filter, a high correlation filter, and principal component analysis (PCA). The linear combination (lincomb) filter finds linear combinations of two or more variables and removes columns to resolve the issue and avoid both collinearity and dimension reduction and it was repeated until the feature set was full rank. The high correlation (corr) filter removes those variable features from the feature set that have a large absolute correlation. A user-specified threshold was chosen to determine the largest allowable absolute correlation. For each pipeline, this threshold was set to 0.6 when using all sequences and 0.8 for the subgroup analyses to retain most important features. By determining the fraction of total variance that should be covered by the components, the number of components retained in the PCA transformation was calculated. The threshold was set at 80% for all sequences and 90% for sub-group analyses, with the intention of preserving enough information to enable model fitting. Feature selection was performed using the recipes package in R version 4.0.2 [34,35]. All features were standardized using the z-score transformation prior to feature selection [21]. In patients with any missing mask (absence of necrotic/edema masks), radiomic features were not calculated, and in those, the missing values were imputed using mean imputation. Additionally, model performance was also evaluated when using all features (full feature set) without a priori feature reduction (using PCA or correlation filter). In models using a full feature set, features were selected through inbuilt (embedded) feature selection of the machine learning models rather than a separate feature selection method like correlation filter or PCA. The estimated number of features used in model fitting after feature selection is provided in the Supplementary Materials (Table S2). 

### 2.8. Model Fitting

Multiple machine-learning predictive models were analyzed to determine the optimal classifier. These models were: linear classifiers (linear, multinomial logistic, ridge, elastic net (enet), and LASSO (least absolute shrinkage and selection operator) regression), non-linear classifiers (neural network, support vector machine with a polynomial kernel (svmPoly), SVM with a radial kernel (svmRad), and multi-layer perceptron (MLP)), and ensemble classifiers (random forest, a generalized boosted regression model (GBRM), and boosting of classification trees with adaBoost).

### 2.9. Classifier Model Performance Evaluation

**Figure 2:**
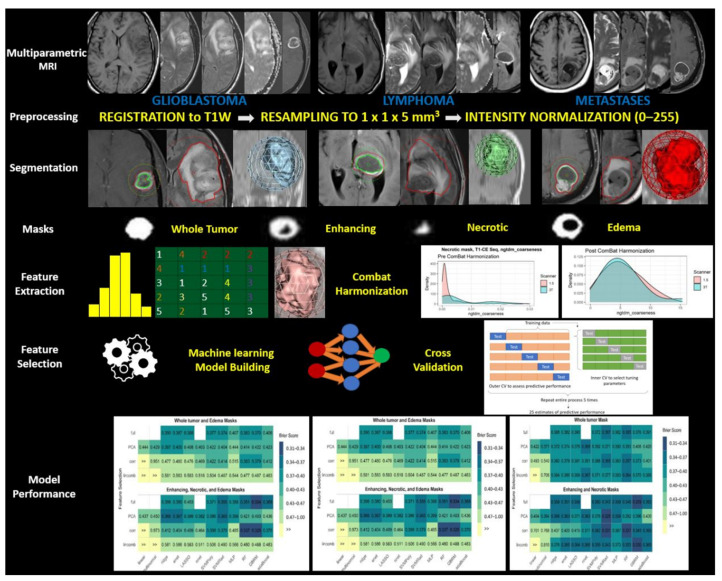
Schematic overview of overall workflow of the study.

All models were fit using the three-feature selection techniques as well as the full-feature set. Three models could not be fit with the full feature set: linear regression and multinomial logistic regression since these did not yield a unique solution secondary to more features than the sample size. In addition, the neural network was too computationally intensive to be fit to the full feature set. Thus, a total of 45 possible model/feature selection combinations were evaluated. These were then analyzed for all of the combined sequences as well as for individual MRI sequences. The predictive performance of each model was evaluated using 5-fold repeated cross-validation. Nested cross-validation was used to tune important parameters to avoid bias from overfitting. Each cross-validated split of the data was used to perform feature selection techniques to avoid bias in the estimate of predictive performance (details in Text S1). The overall workflow is provided in Figure 2.

## 3. Statistical Analysis

**Figure 3:**
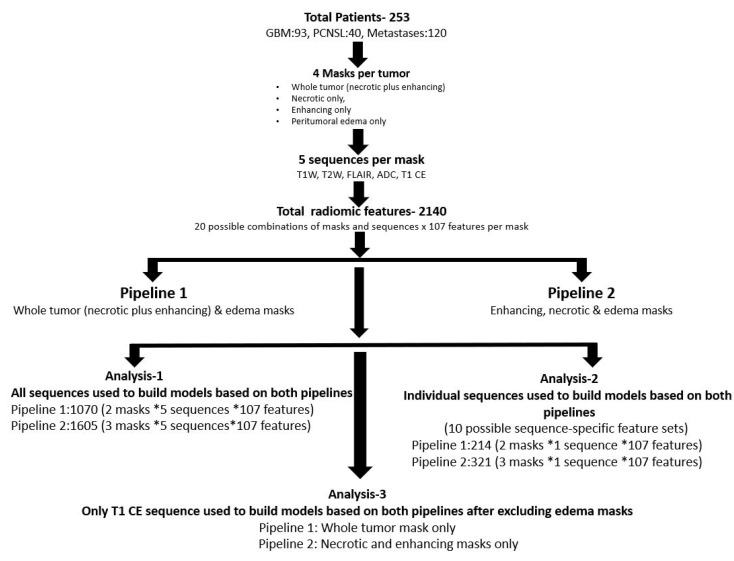
Primary and subgroup analysis workflow of both pipelines.

The data were evaluated using two pipelines. In both pipelines, all five sequences were evaluated. The first pipeline used whole tumor and edema masks and the second used necrotic, enhancing, and edema masks (Figure 3). Since the primary goal was to determine which pipeline performs better in a three-class problem, the radiomics data were split as follows: the first pipeline included 1070 possible features (2 masks × 5 sequences × 107 features), and the second pipeline included 1605 possible features (3 masks × 5 sequences × 107 features). 

Additional analysis was performed to assess best predictive performance amongst individual MRI sequences. This was carried out using the same two pipelines described above, but with each of the five sequences in the feature set individually. In addition, models were also fit only to the T1-CE sequence without the edema masks in both pipelines. 

Predictive performance was rated with Brier score, the categorical analog to mean squared error with lower scores indicating better predictive performance. Paired *t*-tests were performed on the resampled distribution of the Brier scores for the best performing models to evaluate if significant differences in predictive performance existed, with *p*-values adjusted for multiple comparisons using the false discovery rate adjustment [36]. Model fitting and cross-validated predictive performance was implemented using the MachineShop package in R version 4.0.2 [37]. Cross-validated multi-class AUC was also computed using the pROC package in R version 4.0.2 [38]. To provide a measure of the variance for the Brier score, accuracy, and multi-class AUC, confidence intervals were constructed from 1000 bootstrapped samples from the cross-validated estimates. To evaluate the significance of the best performing model, a permutation test was performed using 1000 permutations of the data. The permutation test compares the observed measure of predictive performance (Brier score) to its null distribution, which is obtained by permuting the class labels.

## 4. Results

### 4.1. Patient Characteristics

There were 253 patients (males 128, females 125) in the study population (GBM 93, PCNSL 40, metastases 120). The mean age of the population was 62 ± 11.4 years. The demographic and tumor characteristics are provided in Table 1.

### 4.2. Model Performance

The top-performing model when combining all sequences was GBRM using the high correlation filter (AUC: 0.910; Brier score: 0.325). T1-CE was the best sequence when comparing individual sequences with GBRM using the full feature set, and embedded feature selection showed the highest performance (Brier score: 0.311; AUC: 0.908) (Table 2). The permutation test *p*-value for the GBRM using the full feature set on the T1-CE sequence was 0.0010, which provides strong evidence that this classifier is able to identify a dependency structure in the data to make accurate predictions.

When assessing model performance without the edema mask, the highest prediction performance was obtained using the svmRAD classifier with the PCA feature selection method on the T1-CE sequence (Brier score: 0.325; AUC: 0.894). The paired *t*-test *p*-values were 0.1582, 0.9827, and 0.2540 when comparing all sequences vs. the T1-CE sequence, all sequences vs. the T1-CE sequence without the edema mask, and the T1-CE sequence vs. T1-CE sequence without the edema mask, respectively, indicating no significant differences in predictive performance between these models. Table 3 provides the top five models with the lowest Brier score for these sequence–mask combinations. 

**Figure 4:**
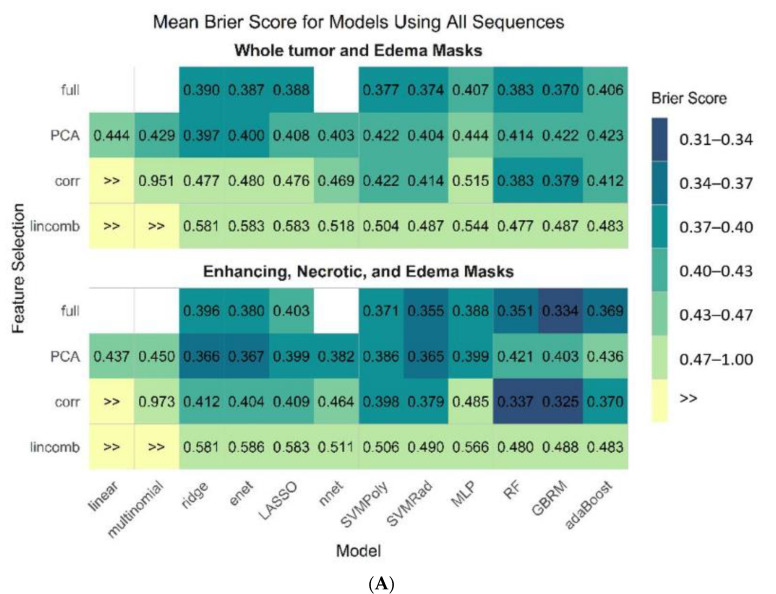

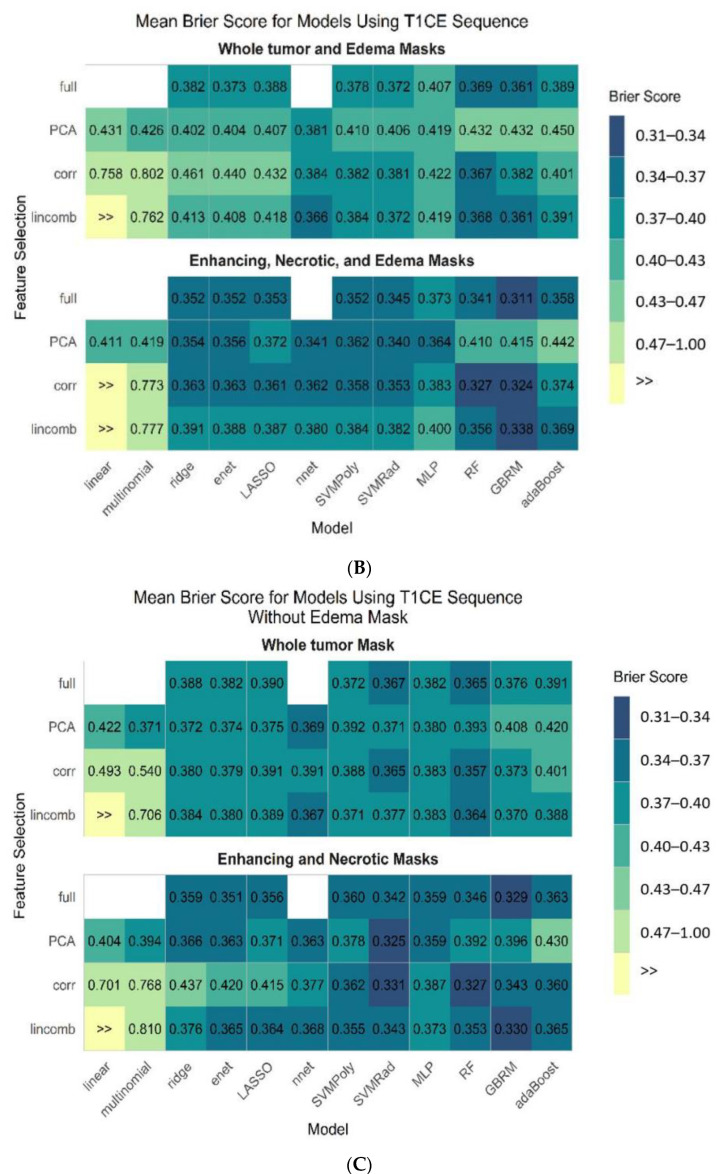
Mean estimate of cross-validated Brier score for all 45 model and feature selection combinations on both pipelines from all sequences (**A**), T1-CE sequence (**B**), and using T1-CE sequence without edema mask (**C**).

Figure 4A–C display the mean estimate of the cross-validated Brier score for all 45 model and feature selection combinations on both pipelines from all sequences, the best performing individual sequence (T1-CE), and the T1-CE sequence without the edema mask, respectively.

### 4.3. Tumor Subregions Performance

The second pipeline (necrotic, edema, and enhancing masks) performed better in all sequence combinations than the first. The cross-validated accuracies for the top three models (GBRM corr, GBRM full, and svmRAD PCA) in the second pipeline were 77, 80, and 78%, respectively, while those of the top three models for the first pipeline (GBRM, GBRM, and RF) were 73, 75%, and 75%, respectively. The predictive performance of both pipelines for all sequence combinations is provided in Table 4 (details in the Supplementary Materials, Tables S3–S5).

### 4.4. Comparison of Predictive Performance between Two Pipelines

The mean difference between the Brier scores for the best models using all sequences on the two pipelines was 0.045 (*p* = 0.0002), indicating that the second pipeline using three separate masks had significantly better predictive performance than the first.

### 4.5. Feature Importance of the Models

Feature importance was computed for the best performing models in three groups (Supplementary Materials, Table S6). For first pipeline, features extracted from whole tumor mask had the highest importance. For the second pipeline, although the necrotic mask had the highest feature importance, the majority of the important features were extracted from the enhancing component. These features were a combination of shape and first- and higher-order texture features.

### 4.6. Confusion Matrix for the Best Performing Model

The confusion matrix was obtained from the cross-validation resamples from the overall best model, which was the GBRM fit to all features from the T1-CE sequence. Overall, the model performed well in classifying the three tumor types. Incorrect predictions tended to favor the tumor types with more patients in the observed data. Metastatic tumors make up the largest percentage of tumors in the observed data (47.4%), and the model correctly classified these tumors 39.1% of the time. Misclassified metastatic tumors are more likely to be classified as GBM compared to PCNSL. PCNSL tumors make up the lowest percentage of tumors in the observed data (15.8%), and the model correctly classified them 9.8% of the time. Misclassified PCNSL tumors are more likely to be classified as metastatic compared to GBM. Finally, GBM tumors make up 36.8% of the observed data, and the model correctly classified them 30.7% of the time. For the misclassified GBM tumors, the model was more likely to predict metastatic tumors compared to PCNSL (Table 5).

## 5. Discussion

Our study evaluated the diagnostic performance of MP-MRI radiomics using various feature selection strategies and machine learning classifiers for a three-class classification problem. We found that using separate masks for tumor sub-components significantly improved the classification performance over using a combined mask for the enhancing and necrotic component with an edema mask. The overall best performing model was the GBRM with embedded feature selection extracted from the T1-CE sequence followed by GBRM with the high correlation extracted from the T1-CE sequence. The performance of the individual T1-CE sequence (without additional edema mask features) was also comparable to that of the best performing models. 

We evaluated twelve classifier models and four feature selection methods. Overall, GBRM and random forest models using embedded feature selection were the best performing models in both pipelines. Both of these models are ensemble classifiers, which build prediction models by combining collections of base learning models—in this case, decision trees. The classifications from many decision trees are aggregated by selecting the class that is predicted most often. Both approaches allow for non-linear relationships of the features in the model and perform embedded feature selection. We also found SVM classifiers using the radial kernel to be among the top-performing models. SVM classifiers incorporate all features and uses projection to perform non-linear classification. The high performance of the RF, GBRM, and SVM classifiers indicates that when using radiomics to differentiate between GBM, PCNSL, and metastases tumors, it is important to utilize machine learning techniques that are flexible enough to incorporate non-linear relationships between the features and tumor classes. 

Our study also demonstrates the variations in the model’s performance based on the combination of machine learning and feature selection techniques. Despite the fact that the models’ performance was comparable to that of some of the top-performing models, the overall differences in model performance, even when using the same mask–sequence combination, calls for a more robust comparison of these techniques to determine the optimal model. This is critical for model generalizability, as reliance on a single model may have limitations for wider adoption into clinical practice [39].

Another important observation was that the best predictive models used embedded feature selection over a priori feature reduction. The high performance of the embedded-type GBRM and random forest classifiers on the full feature set in our study indicates that the loss of information from a priori feature selection methods may be considerable and should not be ignored. Filter selection methods do not incorporate learning, ignore the effects of interaction among features, and only consider noise in the feature. In contrast, embedded classifiers involve feature selection as part of model-building process and identify the suitable feature set as an intrinsic model-building metric during learning. Unlike wrapper methods, model learning is not separated from the feature selection process. Embedded models measure the feature usefulness and account for the interaction of features in a similar manner to that of wrapper methods. However, they are fast, less prone to overfitting, and computationally less intensive than wrapper methods [40]. 

Feature importance showed that the majority of the high-performing features were extracted from the whole tumor mask for the first pipeline; for the second pipeline, the top-ranked feature was extracted from the necrotic mask. However, for the second pipeline, the majority of the top-ranked features were from the enhancing mask followed by the necrotic mask. There was no contribution of edema masks for any of the top-ranked features. This again highlights the fact that performance of T1-CE without the edema mask was similar to that of T1-CE with the edema mask and multiparametric MRI. Furthering our understanding of the biological correlates of these features remains a work in progress. However, a combination of different radiomic features (first-order, second-order, and shape features) was seen among the top-performing features. This reemphasizes that different radiomic features may carry different tumoral information, and, thus, inclusion of multiple feature types may improve the prediction performance over just first-order features. This may be especially true for GBM in which there is significant intra-tumor heterogeneity [41].

The comparable performance of T1-CE-derived models to those using MP-MRI is noteworthy, as the T1-CE sequence is universally performed, and radiomics analysis of a single sequence and less masks (enhancing and necrotic only) is less resource intensive and time efficient and may be a more robust approach for integration into clinical workflow. The comparable predictive performance of T1-CE-based models has also been shown previously for glioma grading [42] and survival [43]. 

To date, very few studies have addressed this three-class problem using radiomics. Di Ieva et al. [44] utilized fractal analysis as a quantitative tool to differentiate among multiple brain tumor types and found significant difference between lymphoma and high-grade glioma but not metastases. Their study had a small patient population (*n* = 78) and utilized a single quantitative feature (fractal dimension) extracted from the T1-CE sequence only. Ma et al. [45] used whole-tumor histogram analysis of normalized cerebral blood volume to differentiate between GBM, PCNSL, and brain metastases. However, their study analysis showed only two-class classification results (GBM versus PCNSL, GBM versus metastases, and PCNSL versus metastases), and no three-class classification was performed. Our approach is more pragmatic, as using only a two-class approach may introduce a selection bias and overestimate the classification accuracy.

There are prior non-radiomic studies that have addressed the three-class problem classification. The majority of them used advanced imaging sequences like perfusion imaging [46], arterial spin labelling [47], spectroscopy [48,49], diffusion tensor imaging [50,51], or susceptibility weighted imaging [52]. Most of these techniques are complex, are not universally performed, increase scan time, and require expert evaluation; thus, they are limited in generalizability. In contrast, our approach analyzed conventional MP-MRI sequences that are performed routinely at all institutions.

Besides the limitations of retrospective data, our study lacked an external validation group to improve the generalizability of the optimal model. However, we did perform nested cross-validation to avoid bias and validated our models. Secondly, we did not assess deep learning-based models in our study, and their impact on three-class classification problems remains undefined. We also could not evaluate the impact of genomic variations (isocitrate dehydrogenase and O6-methylguanine-DNA methyltransferase promoter methylation (MGMT)) due to the lack of such information in several GBM patients. Lastly, we only selected metastatic tumors with known lung or breast primary. The inclusion of only these two metastatic tumor types in our study cohort may have introduced selection bias. While these are the two most common brain metastases, it is possible that adding further sub-types of metastases may decrease the overall model performance and affect model generalizability. However, this study is an improvement in terms of patient selection compared to prior radiomic studies and reflects a more comprehensive patient population encountered in clinical practice. 

## 6. Conclusions

Our results show that a three-class problem can be addressed with excellent diagnostic performance using a radiomics-based approach. Additionally, the choice of appropriate feature selection and machine learning techniques needs to be more robust since it can have a significant impact on model performance. Overall, the models developed with separate enhancing and necrotic masks significantly outperform those where the two components were treated as a single mask. Finally, radiomic features derived from the T1-CE sequence performed similarly to MP-MRI-based models for this specific problem.

## Figures and Tables

**Table 1:** Patient demographics and tumor characteristics.

Demographics	GBM	PCNSL	Metastases
Patients (253)	93	40	120; breast (29);
			lung (91)
Age in years (mean ± SD)	62 ± 11	62 ± 13	62 ± 10
Gender			
Male	52	22	54
Female	41	18	66
Localization			
Supratentorial	91	33	Breast (17); lung (62)
Infratentorial	2	4	Breast (6); lung (14)
Both	0	3	Breast (6); lung (15)
Multiplicity			
Single	83	19	Breast (21); lung (64)
Two	5	8	Breast (2); lung (9)
≥Two (multiple)	5	13	Breast (6); lung (18)
Necrosis			
Yes	92	10	Breast (19); lung (68)
No	1	30	Breast (10); lung (23)

**Table 2:** Predictive performance of individual MRI sequences.

	Whole Tumor and Edema Masks					Necrotic, Enhancing, and Edema Masks				
Sequence	Model	Feature Selection	Brier Score Mean (95% CI)	Accuracy Mean (95% CI)	*p*-Value	Model	Feature Selection	Brier Score Mean (95% CI)	Accuracy Mean (95% CI)	*p*-Value
T1-CE	gbrm	full	0.361 (0.222, 0.528)	0.756 (0.660, 0.863)	-	gbrm	full	0.311 (0.223, 0.466)	0.796 (0.667, 0.880)	-
T1W	gbrm	full	0.405 (0.292, 0.553)	0.735 (0.620, 0.863)	0.0028	gbrm	full	0.340 (0.231, 0.463)	0.771 (0.680, 0.900)	0.0155
T2W	rf	corr	0.381 (0.280, 0.481)	0.730 (0.660, 0.804)	0.1582	gbrm	corr	0.340 (0.224, 0.506)	0.772 (0.608, 0.863)	0.0216
ADC	rf	lincomp	0.420 (0.320, 0.520)	0.705 (0.600, 0.784)	0.0002	gbrm	corr	0.349 (0.197, 0.505)	0.756 (0.686, 0.843)	0.0034
FLAIR	rf	full	0.418 (0.334, 0.511)	0.699 (0.608, 0.765)	<0.0001	gbrm	full	0.353 (0.242, 0.479)	0.768 (0.680, 0.863)	0.0092

gbrm: gradient boost regression model; rf: random forest; full: full feature set; corr: high correlation filter; lincomb: linear combination filter.

**Table 3:** Top five models with the lowest Brier score for models using all sequence and mask combinations.

**Using All (Multiparametric MRI) Sequences**												
**Rank**	**Masks**	**Model**	**Feature Selection**		**Mean Brier**		**95% CI** **Brier**		**Mean** **Multi-AUC**		**95% CI** **Multi-AUC**	
1	N, E, edema	gbrm	corr		0.325		(0.232, 0.488)		0.910		(0.833, 0.959)	
2	N, E, edema	gbrm	full		0.334		(0.215, 0.434)		0.900		(0.832, 0.963)	
3	N, E, edema	rf	corr		0.337		(0.269, 0.455)		0.899		(0.805, 0.948)	
4	N, E, edema	rf	full		0.351		(0.278, 0.466)		0.893		(0.819, 0.962)	
5	N, E, edema	svmRad	full		0.355		(0.259, 0.468)		0.878		(0.762, 0.947)	
**Using T1-CE Sequence**												
**Rank**	**Masks**	**Model**		**Feature Selection**		**Mean Brier**		**95% CI** **Brier**		**Mean** **Multi-AUC**		**95% CI** **Multi-AUC**
1	N, E, edema	gbrm		full		0.311		(0.223, 0.466)		0.908		(0.820, 0.959)
2	N, E, edema	gbrm		corr		0.324		(0.229, 0.430)		0.904		(0.841, 0.964)
3	N, E, edema	rf		corr		0.327		(0.265, 0.451)		0.907		(0.808, 0.954)
4	N, E, edema	gbrm		lincomb		0.338		(0.225, 0.541)		0.892		(0.797, 0.950)
5	N, E, edema	svmRad		PCA		0.340		(0.253, 0.443)		0.894		(0.824, 0.955)
**Using T1-CE Sequence without Edema Mask**												
**Rank**	**Masks**	**Model**		**Feature Selection**		**Mean Brier**		**95% CI** **Brier**		**Mean** **Multi-AUC**		**95% CI** **Multi-AUC**
1	N, E	svmRad		PCA		0.325		(0.255, 0.485)		0.894		(0.255, 0.485)
2	N, E	rf		corr		0.327		(0.261, 0.458)		0.905		(0.261, 0.458)
3	N, E	gbrm		full		0.329		(0.230, 0.473)		0.902		(0.230, 0.473)
4	N, E	gbrm		lincomb		0.330		(0.219, 0.446)		0.901		(0.219, 0.446)
5	N, E	svmRad		corr		0.331		(0.237, 0.425)		0.895		(0.237, 0.425)

N: necrotic mask; E: enhancing mask; gbrm: generalized boosted regression model; rf: random forest; svmRad: SVM with a radial kernel; corr: high correlation filter; full: full feature set; lincomb: linear combination filter; PCA: principal component analysis.

**Table 4:** Predictive performance of both pipelines for all sequence combinations.

Sequence	Whole Tumor and Edema Masks				Necrotic, Enhancing, and Edema Masks			
	Model	Feature Selection	Brier Score Mean (95% CI)	Accuracy Mean (95% CI)	Model	Feature Selection	Brier Score Mean (95% CI)	Accuracy Mean (95% CI)
All sequences	gbrm	full	0.370 (0.236, 0.460)	0.732 (0.627, 0.824)	gbrm	corr	0.325 (0.232, 0.488)	0.771 (0.608, 0.843)
T1-CE	gbrm	full	0.361 (0.222, 0.528)	0.756 (0.660, 0.863)	gbrm	full	0.311 (0.223, 0.466)	0.796 (0.667, 0.880)
T1-CE without edema mask	rf	corr	0.357 (0.262, 0.443)	0.752 (0.620, 0.843)	svmRad	PCA	0.325 (0.255, 0.485)	0.782 (0.686, 0.860)

gbrm: gradient boost regression model; rf: random forest; full: full feature set; corr: high correlation filter; svmRad: SVM with a radial kernel; PCA: principal component analysis.

**Table 5:** Confusion matrix for the best performing model (GBRM fit using the full feature).

	Observed Tumor Type			
Predicted	Metastatic	PCNSL	GBM	Total
**Metastatic**	39.1%	4.5%	5.1%	48.7%
**PCNSL**	2.5%	9.8%	1.0%	13.3%
**GBM**	5.8%	1.5%	30.7%	38.0%
**Total**	47.4%	15.8%	36.8%	100%

PCNSL: primary CNS lymphoma; GBM: glioblastoma; GBRM: generalized boosted regression model.

## Data Availability

The datasets generated during and/or analyzed during the current study are available from the corresponding author on reasonable request.

## Supplementary Materials

The following are available online at https://www.mdpi.com/article/10.3390/cancers13112568/s1, Text S1, Figure S1. Block diagram showing mask separation, Table S1. Least axis length masks, Table S2. Estimated number of features used in model fitting after feature selection was performed, Table S3. Predictive performance of the top 10 models in terms of mean cross-validated Brier Score and AUC for models across all sequences, Table S4. Predictive performance of the top 10 models in terms of mean cross-validated Brier Score and AUC for models across individual sequences, Table S5. Predictive performance of the top 10 models in terms of mean cross-validated Brier Score and AUC for models on T1 CE sequence without edema mask, Table S6. Feature importance for the highest performing models.
