# Supplementary material for: Radiomic Based Machine Learning Performance for a Three Class Problem in Neuro-Oncology: Time to Test the Waters?
Source: Cancers (Basel). 2021 May 24;13(11):2568. doi: 10.3390/cancers13112568 (PMC8197204; doi:10.3390/cancers13112568)
Supplement: Supplementary file 1 [file cancers-13-02568-s001.zip › cancers-1132948-supplementary .pdf]

Supplementary Materials: Radiomic Based Machine Learning Performance for a Three Class Problem in Neuro-Oncology: Time to Test the Waters?

Sarv Priya, Yanan Liu, Caitlin Ward, Nam H. Le, Neetu Soni, Ravishankar Pillenahalli Maheshwarappa, Varun Monga, Honghai Zhang, Milan Sonka and Girish Bathla

Text S1

1. Patient Selection

This was a retrospective study approved by the local institutional review board (IRB-ID 201912239). Between 2010–2019, patients above the age of 18 years were identified using a combination of electronic medical records and institutional cancer registries. Since lung and breast cancer account for most of the cases of brain metastases, the metastatic lesion cohort was confined to patients with a known lung or breast primary. Eligibility criteria included: preoperative MRI scans that had all multiparametric (axial T1W, T2W, FLAIR, ADC and T1 contrast enhanced (CE)) sequences available, presence of a contrast enhancing tumor, and no prior history of treatment, biopsy or surgical resection. Patients with non-enhancing tumors and motion artifact were excluded. A total of 253 patients were included in the study (metastatic (n = 120, 47.4%), PCNSL (n = 40, 15.8%), and GBM (n = 93, 36.8%)).

2. MRI Imaging Parameters

2.1. MRI Scanning Parameters for Siemens 1.5 T MRI (Siemens, Erlangen, Germany)

T1W (TR/TE/TI: 1950/10/840, NEX: 2, slice thickness: 5 mm, matrix: 320 × 256, field of view 240 mm, pixel size 0.75 mm)

T2W (TR/TE: 4000/90, NEX: 2, slice thickness: 5 mm, matrix: 512 × 408, field of view 240 mm, pixel size 0.5 mm);

FLAIR (TR/TE/TI: 9000/105/2500, NEX: 1, slice thickness: 5 mm, matrix: 384 × 308, field of view 240 mm, pixel size 0.6 mm)

DWI (TR/TE: 4000/74, NEX: 3, slice thickness: 5 mm, matrix: 128 × 128, field of view 240 mm, pixel size 1.8 mm)

T1W-CE (TR/TE/TI: 570/13, NEX: 2, slice thickness: 5 mm, matrix: 384 × 312, field of view 240 mm, pixel size 0.62 mm)

T1W-CE images were acquired 2–3 minutes after administration of gadobenate dimeglumine (Multihance; Bayer Healthcare Pharma) or gadobutrol (Gadavist; Bayer Healthcare Pharma, Berlin, Germany) injected at the rate of 0.1 mL/kg body weight.

2.2. MRI Scanning Parameters for Siemens 3 T MRI (Siemens, Erlangen, Germany)

T1W (TR/TE/TI: 2000/11/899, NEX: 1, slice thickness: 5 mm, matrix: 384 × 312, field of view 240 mm, pixel size 0.62 mm)

T2W (TR/TE: 4000/105, NEX: 2, slice thickness: 5 mm, matrix: 448 × 364, field of view 240 mm, pixel size 0.5 mm)

FLAIR (TR/TE/TI: 9000/108/2500, NEX: 1, slice thickness: 5 mm, matrix: 384 × 312, field of view 240 mm, pixel size 0.62 mm)

DWI (TR/TE: 4250/64, NEX: 1, slice thickness: 5 mm, matrix: 160 × 160, field of view 240 mm, pixel size 1.5 mm)

T1W-CE (TR/TE/TI: 2000/12/900, NEX: 1, slice thickness: 5 mm, matrix: 384 × 312, field of view 240 mm, pixel size 0.62 mm)

3. Image Preprocessing Was Performed as per International Biomarker Standardiza-Tion Initiative (IBSI) Guidelines with Following Steps Performed:

Resampling—Registration—Normalization—Segmentation—Feature Extraction—Model Building

Image Preprocessing:

1. Image format conversion: DICOM images were converted to NIfTI
2. Image resampling: To standardize MR image resolution, all MR image volumes were resampled to 1 × 1 × 5 mm^3 voxel size using the AFNI package (https://afni.nimh.nih.gov/).
3. Co-registration: Images acquired during the same scanning session were mutually registered to T1 W sequence using Advanced Normalization Tools (ANTs) (http://stnava.github.io/ANTs/).
4. Intensity normalization: Image intensities were normalized to [0,255] using the feature scaling method available in the ANTs registration suite (http://stnava.github.io/ANTs/).

4. Tumor Segmentation

1. Highly accurate 3D segmentation was performed on axial T1-CE and FLAIR images by a semi-automated approach using LOGISMOS (Layered Optimal Graph Image Segmentation for Multiple Objects and Surfaces) software. After the automated LOGISMOS graph segmentation, the next step allows the expert observer to efficiently fix small localized segmentation inaccuracies by interacting with the LOGISMOS optimization algorithm instead of redrawing surfaces on all affected 2D slices. As such, only a few additional expert-identified points indicating correct surface locations are typically needed to modify the 3D surfaces and thus correct the 3D segmentations in their entirety.
2. Region of interests/masks generation- Four different region of interests (masks) were created using T1-CE and FLAIR images: i) whole tumor (enhancing plus necrotic); ii) enhancing only; iii) necrotic only; and iv) peritumoral edema. The first three masks were created from the T1-CE images by first generating masks for the whole tumor (encompassing both enhancing and necrotic segments) and necrotic components and then subtracting the necrotic mask from whole tumor to generate enhancing mask. The FLAIR images were used to generate a mask for the entire lesion (whole tumor and surrounding edema). Edema mask was eventually obtained by subtracting T1-CE derived whole tumor mask from the FLAIR mask (Figure S1).

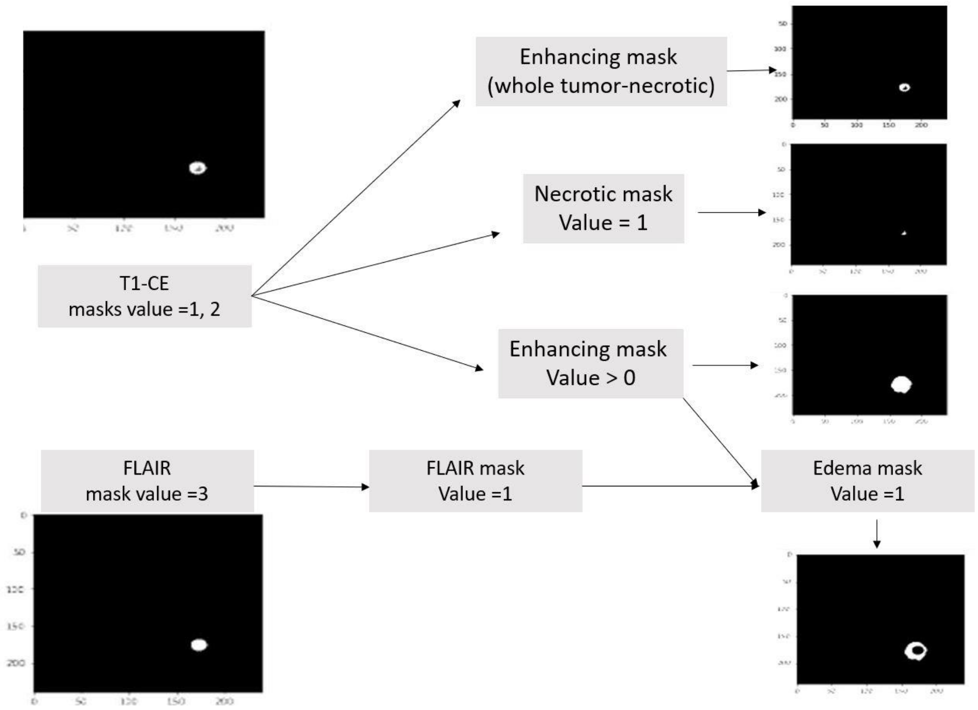

Figure S1. Block diagram showing mask separation. The left two masks were the masks segmented using LOGISMOS software. Different masks had different values because we used Logismos software to segment out the masks. In order to separate different masks, the software assigned different masks different value. The right 4 figures were separated masks: all the masks value equals 1 and background equals 0. We used the right separated masks to extract radiomic features.

5. Texture Feature Extraction

1. A total of 107 features were extracted using Pyradiomics 3.0:3D shape features (n = 14), first order features (n = 18), gray level co-occurrence matrix features (n = 24), gray level dependency matrix features (n = 14), gray level run length matrix features (n = 16), gray level size zone matrix features (n = 16) and neighboring gray tone difference matrix features (n = 5).
2. The default value for the number of bins was fixed by bin width of 25 gray levels. In rare cases where the edema or necrosis were minimal, leading to absence of a corresponding mask, the value of the corresponding feature was set to −9999.

Table S1. Least axis length masks.

Patient	Edema Mask (3D Shape)	Whole Tumor Mask (3D Shape)	Enhancing Mask (3D Shape)	Necrotic Mask (3D Shape)
**Patient Number**	**Least Axis Length (mm)**	**Least Axis Length (mm)**	**Least Axis Length (mm)**	**Least Axis Length (mm)**
1	18.7	27.2	27.2	−10,000
2	25.5	15.4	15.4	−10,000
3	81.8	33.9	32.8	16.5
4	28.8	16.5	15.3	8.87
5	23.7	13.1	12.8	0
6	25.4	9.23	9.23	−10,000
7	17.6	11	11	−10,000
8	39.3	33.2	30.6	21.9
9	58.1	19.2	18.8	9.05
10	35.9	19.8	19.4	6.17
11	19.6	9.93	9.94	0
12	−10,000	−10,000	−10,000	−10,000
13	8.26	8.22	8.22	−10,000

14	46.4	25.4	25.4	−10,000
15	57.5	28.1	28	7.59
16	37	23.5	23.5	−10,000
17	35.7	21.2	21.2	−10,000
18	45.2	8.84	34.2	32.7
19	12.5	−10,000	6.99	6.99
20	33.4	8.4	9.57	0
21	9.41	8.85	8.85	−10,000
22	47	18.2	18	3.97
23	23.9	15	15	−10,000
24	5.61	0	0	−10,000
25	10.3	9.24	8.88	0
26	17.2	13.3	12.4	6.54
27	55.6	17.2	20.8	17.7
28	31.7	19.3	19.3	−10,000
29	48.5	20.1	19.6	6.27
30	27.8	23.1	20.4	13.9
31	36.9	48.3	47.2	23
32	51.6	39.3	38.2	15.3
33	67.6	29.9	29.3	11.3
34	52.2	38.1	37.7	23.7
35	42.2	25.2	23.9	11.3
36	42	57	51	32.1
37	50.7	37.3	34.1	22.6
38	46.8	28.3	26.8	17.1
39	43.3	54.8	51.1	28.1
40	48.1	21.5	21	9.78
41	60.4	35.6	34.2	22.8
42	61	37.4	35.7	15.8
43	50.4	47	40.7	30.8
44	36.7	34	31.6	20.3
45	71.5	31	30.4	15.1
46	57.5	44.1	39.8	23.4
47	45.3	29.8	27.6	17.4
48	57.5	37.4	34.5	24.5
49	16.3	11.2	10.9	5.31
50	26.7	28	26.9	9.28
51	33.3	23.4	22.3	11.5
52	49.2	45.9	42.4	21.6
53	40.7	45.2	41.3	25.1
54	40.8	30.8	29.1	11
55	37.9	12.7	12.6	0
56	38.1	15.2	14.8	5
57	34.9	26	24.3	12
58	52.4	45.4	44.8	14
59	56.9	29.8	28.5	16.7
60	63.8	38.6	36.6	21.4
61	50.8	24.9	25.8	10.9
62	38.9	29.4	28.8	11.5
63	38.3	21.7	20.6	11.1
64	61	29.4	28.5	11.5
65	55.9	24.8	24.2	11.2
66	63.2	31.6	32.1	17.1
67	40.1	24.2	22.2	14.4
68	46	24.8	23.6	13.1
69	58.3	25.1	24.1	10.3

70	44.5	21	20.1	10.5
71	50.7	37.5	35.9	19.2
72	25.6	21.3	21.2	3.74
73	46.7	28.3	26.3	16.3
74	46	35.2	34.9	27.4
75	49.1	42.4	38.3	26.5
76	45.9	41	36.7	28.1
77	56.8	29.6	28.3	20.7
78	49.9	27.1	26	13.6
79	52.1	34.3	34.3	−10,000
80	45.6	41.8	41.8	6.37
81	40.6	36.7	35.9	25
82	39.6	44.9	43.2	18.5
83	61.9	35	32.8	19.6
84	49.3	34.4	32.7	17.6
85	41.2	30.2	27	17.9
86	41.6	27.9	27.4	8.16
87	50.2	39.2	35.9	19.7
88	48.1	32.7	30.5	25.8
89	55.2	45.1	42.9	20.1
90	35.7	36.1	35	20.4
91	22.1	15.8	14.9	8.96
92	24.8	22.7	21.4	10.3
93	54.6	35.8	35.6	7.23
94	46.7	34.5	32.7	19.1
95	40	37.1	35.8	15.6
96	27.3	16	15.3	6.86
97	23.4	19.7	19.7	−10,000
98	48.1	24.6	23.7	12.3
99	45.2	29.7	27.4	13.6
100	58.1	49	48.8	5.21
101	54	40.6	38.6	22.8
102	32.3	25.3	23.8	11.8
103	59	38	37.7	8.8
104	22	20.9	20.5	5.96
105	48.8	23.4	22.1	13.4
106	50.8	30.6	30.1	10.1
107	48	38.5	36.7	20.5
108	36.5	28	26	15.8
109	39.5	29.7	28.4	19.8
110	22.5	14.3	13.5	0
111	38.8	20.9	20.6	6.49
112	33	34.8	33.7	13.7
113	44	36.5	33	22.6
114	36.9	34.5	34.4	5.03
115	50.5	50.7	48.2	26.4
116	30.5	19.1	18.3	8.67
117	48.2	41.6	37.4	26.4
118	50.6	41.2	37.9	25.5
119	41.4	38.2	37.1	15.8
120	80.8	49.1	49.4	22.5
121	43	43.9	44	14.6
122	38.1	17.7	17.3	5.86
123	31.7	17.8	17.8	−10,000
124	11.6	15.3	14.2	6.31
125	19.5	14.3	12.9	7.54

126	9.25	0	0	−10,000
127	30.1	18.6	17.2	8.38
128	16.5	9.5	9.5	−10,000
129	26.9	12	11.7	0
130	17	0	0	−10,000
131	0	5.81	5.81	−10,000
132	50.2	13.5	13.5	−10,000
133	24.7	22.3	20.4	10.8
134	44.8	24.2	24.2	−10,000
135	24.8	12.1	12.1	−10,000
136	37.6	9.33	9.51	0
137	11.9	6.18	6.18	−10,000
138	15.6	15.2	14.6	8.2
139	0	0	0	−10,000
140	35.5	13.9	13.7	0
141	38.8	13.1	12.8	0
142	32.6	10.9	10.6	0
143	52.9	16.1	15.6	6.06
144	12.8	0	0	−10,000
145	13.9	10.2	10.2	−10,000
146	43.4	7.87	24.3	21.2
147	9.58	6.99	6.99	−10,000
148	35.7	16.6	16.2	5.36
149	48.7	18.1	16.9	9.81
150	36.7	16.6	15.2	9.54
151	36.4	7	19.1	19.4
152	27.3	16.4	16.4	−10,000
153	25	11.8	11.8	−10,000
154	25.2	10.3	10.3	−10,000
155	37.2	23.9	22.4	11.6
156	18.4	9.79	9.51	4.24
157	34.3	28.2	28	4.79
158	47.3	17.1	16.5	8.15
159	30.7	23.8	21.8	15
160	42.3	18.7	18.1	5.49
161	29.5	12.8	12.2	5.57
162	7.92	9.29	9.29	−10,000
163	37.3	14.3	14	0
164	54.8	13	12.9	0
165	51.9	22	22	−10,000
166	32.2	14.8	19.8	12
167	79.1	20.4	20.3	5.3
168	49.1	22.8	33.6	32.2
169	23.2	21.5	20.5	10.5
170	26.9	17.3	16.6	7.78
171	15	5.93	5.93	−10,000
172	37.7	8.88	8.69	0
173	3.89	9.1	8.82	2.91
174	42	17.6	16.5	8.87
175	80.9	31.3	29.6	13
176	13.9	11.4	11.4	−10,000
177	15.6	13.8	13.5	0
178	32.7	22.1	22.1	−10,000
179	33.9	21.9	21.8	4.27
180	47.6	44.2	37	28.7
181	34.7	14.5	14	9.13

182	9.36	0	0	0
183	30.1	12.6	11.9	0
184	20.6	14.6	14.6	−10,000
185	45.7	39.8	34.8	21.4
186	11.9	12.5	12.1	0
187	6.66	9.04	8.46	0
188	10	9.97	9.97	−10,000
189	20.7	19.5	18.4	12.3
190	29.8	18.3	18.3	−10,000
191	20	12.9	12.5	0
192	22.5	10.3	10.1	0
193	55	31.7	31.2	9.85
194	24.8	9.89	9.89	−10,000
195	7.95	10.9	10.7	4.71
196	14	5.64	5.64	−10,000
197	24.6	9.32	9.32	−10,000
198	54.4	56.7	47.1	38.4
199	33.8	20.9	20	6.84
200	22	16.4	16.1	4.3
201	26.2	14.1	13.8	3.7
202	75.8	30	28.3	13.9
203	14.9	11	10.7	0
204	27.5	28.6	25.2	17.7
205	12.3	12.4	12.1	0
206	6.18	6.8	6.8	−10,000
207	55.7	35	32.1	19.1
208	11.6	10.2	10.2	−10,000
209	27.6	10.9	10.4	4.89
210	17.6	10.6	10.6	−10,000
211	26	17.1	16.5	8.7
212	22.6	14	14	−10,000
213	32.6	15.2	14.8	8.61
214	36.6	22.2	22.2	−10,000
215	−10,000	−10,000	−10,000	−10,000
216	54.6	29.9	29.9	−10,000
217	43	17.2	17.2	−10,000
218	45	22.4	22.4	−10,000
219	24.6	22.2	22.2	−10,000
220	53.1	17.1	16.8	5.29
221	29.6	14.6	14.6	−10,000
222	12.5	11.9	11.9	−10,000
223	40.7	16.2	16.2	−10,000
224	39.5	18.9	18.9	−10,000
225	36.2	19.6	19.6	−10,000
226	62.6	36.9	36.8	0
227	68.4	21	21	−10,000
228	12.1	13.3	13.3	−10,000
229	43.8	25.4	25.4	−10,000
230	34.6	18.3	18.3	−10,000
231	28	14.8	14.8	−10,000
232	47.5	16.2	16.2	−10,000
233	21.4	11.1	11.1	−10,000
234	20.2	13.2	13.2	−10,000
235	20.7	13.8	13.8	−10,000
236	29.5	16.1	16.1	−10,000
237	35.1	14.8	14.8	−10,000

238	48.2	21	21	−10,000
239	−10,000	−10,000	−10,000	−10,000
240	−10,000	−10,000	−10,000	−10,000
241	16.5	12.5	12.5	−10,000
242	27.6	20.2	20.6	13.6
243	41.7	19.4	19.4	−10,000
244	40.1	27.2	27.2	−10,000
245	33.7	20.2	20.2	−10,000
246	54.1	29.2	29.2	8.85
247	27.5	19.4	19.4	−10,000
248	11.9	8.72	8.72	−10,000
249	37.3	22.2	22.2	−10,000
250	36.9	15.5	15.5	−10,000
251	26	19.7	19.7	−10,000
252	45.1	28.4	26.4	17
253	49.1	27	26.9	2.27

Cell number <4 = Patients with less than 4 voxels. Cell number −10,000 = No radiomic features calculated due to absence of corresponding masks.

Table S2. Estimated number of features used in model fitting after feature selection was performed.

	Full	Corr	Lincomb	PCA
**Whole Tumor and Edema Masks**				
All features	1070	120	250	25
Individual Sequences	214	77	210	22
CE without edema	107	36	105	12
**Necrotic, Enhancing, and Edema Masks**				
All features	1605	190	250	35
Individual Sequences	321	115	248	30
CE without edema	214	73	210	22

Note: Estimates for the high correlation filter, linear combinations filter, and PCA may vary between different cross validated split of data and when applied to the individual sequences may vary depending on which sequence was used. CE: Contrast enhanced; Full: full feature set; corr: high correlation filter; Lincomb: linear combinations filter; PCA: Principal component analysis.

Table S3. Predictive performance of the top 10 models in terms of mean cross-validated Brier Score and AUC for models across all sequences.

Rank	Whole Tumor and Edema Masks (First Pipeline)				Necrotic, Enhancing, and Edema Masks (Second Pipeline)			
	**Model**	**Feature Selection**	**Brier Score Mean (95% CI)**	**Accuracy Mean (95% CI)**	**Model**	**Feature Selection**	**Brier Score Mean (95% CI)**	**Accuracy Mean (95% CI)**
**1**	gbrm	full	0.370 (0.236, 0.460)	0.732 (0.627, 0.824)	gbrm	corr	0.325 (0.232, 0.488)	0.771 (0.608, 0.843)
**2**	svmRad	full	0.374 (0.310, 0.460)	0.727 (0.647, 0.824)	gbrm	full	0.334 (0.215, 0.434)	0.776 (0.700, 0.880)
**3**	svmPoly	full	0.377 (0.302, 0.451)	0.745 (0.686, 0.843)	rf	corr	0.337 (0.269, 0.455)	0.785 (0.686, 0.880)
**4**	gbrm	corr	0.379 (0.254, 0.482)	0.733 (0.627, 0.824)	rf	full	0.351 (0.278, 0.466)	0.759 (0.627, 0.920)
**5**	rf	full	0.383 (0.306, 0.453)	0.722 (0.647, 0.804)	svmRad	full	0.355 (0.259, 0.468)	0.753 (0.667, 0.843)
**6**	rf	corr	0.383 (0.299, 0.459)	0.730 (0.620, 0.824)	svmRad	pca	0.365 (0.273, 0.461)	0.732 (0.647, 0.824)
**7**	enet	full	0.387 (0.284, 0.458)	0.727 (0.588, 0.843)	ridge	pca	0.366 (0.290, 0.455)	0.741 (0.647, 0.880)
**8**	lasso	full	0.388 (0.286, 0.454)	0.728 (0.620, 0.824)	enet	pca	0.367 (0.274, 0.481)	0.734 (0.667, 0.804)
**9**	ridge	full	0.390 (0.257, 0.480)	0.729 (0.660, 0.863)	ada	full	0.369 (0.274, 0.479)	0.760 (0.647, 0.804)
**10**	ridge	pca	0.397 (0.288, 0.488)	0.73 (0.647, 0.863)	ada	corr	0.37 (0.325, 0.421)	0.764 (0.608, 0.840)

gbrm—generalized boosted regression model; svmRad—SVM with a radial kernel; svmPoly—support vector machine (SVM) with a polynomial kernel; Enet—elastic net; rf–random forest; lasso—least absolute shrinkage and selection operator; ada—adaboost; full—full feature set; corr—high correlation filter; pca—principal component analysis.

Table S4. Predictive performance of the top 10 models in terms of mean cross-validated Brier Score and AUC for models across individual sequences.

**Rank**	**Whole Tumor and Edema Masks (First Pipeline)**					**Necrotic, Enhancing and Edema Masks (Second Pipeline)**				
	**Seq**	**Model**	**Feature Selection**	**Brier Score Mean (95% CI)**	**Accuracy Mean (95% CI)**	**Seq**	**Model**	**Feature Selection**	**Brier Score Mean (95% CI)**	**Accuracy Mean (95% CI)**
**1**	CE	gbrm	full	0.361 (0.222, 0.528)	0.756 (0.660, 0.863)	CE	gbrm	full	0.311 (0.223, 0.466)	0.796 (0.667, 0.880)
**2**	CE	gbrm	lincomb	0.361 (0.237, 0.486)	0.750 (0.640, 0.824)	CE	gbrm	corr	0.324 (0.229, 0.430)	0.791 (0.686, 0.860)
**3**	CE	nnet	lincomb	0.366 (0.279, 0.463)	0.746 (0.640, 0.863)	CE	rf	corr	0.327 (0.265, 0.451)	0.798 (0.686, 0.900)
**4**	CE	rf	corr	0.367 (0.288, 0.460)	0.740 (0.640, 0.824)	CE	gbrm	lincomb	0.338 (0.225, 0.541)	0.780 (0.686, 0.902)
**5**	CE	rf	lincomb	0.368 (0.288, 0.470)	0.749 (0.608, 0.863)	T1	gbrm	full	0.340 (0.231, 0.463)	0.771 (0.680, 0.900)
**6**	CE	rf	full	0.369 (0.302, 0.472)	0.753 (0.627, 0.863)	T2	gbrm	corr	0.340 (0.224, 0.506)	0.772 (0.608, 0.863)
**7**	CE	svmRad	lincomb	0.372 (0.294, 0.487)	0.726 (0.640, 0.863)	CE	svmRad	pca	0.340 (0.253, 0.443)	0.769 (0.627, 0.860)
**8**	CE	svmRad	full	0.372 (0.286, 0.495)	0.726 (0.640, 0.863)	CE	rf	full	0.341 (0.268, 0.461)	0.760 (0.608, 0.860)
9	CE	enet	full	0.373 (0.301, 0.491)	0.743 (0.647, 0.820)	CE	nnet	pca	0.341 (0.251, 0.427)	0.779 (0.667, 0.860)
10	CE	svmPoly	full	0.378 (0.296, 0.481)	0.720 (0.647, 0.824)	T2	rf	corr	0.342 (0.242, 0.514)	0.771 (0.686, 0.860)

CE—T1 contrast enhanced; gbrm—generalized boosted regression model; nnet—neural network; rf—random forest; svmRad—SVM with a radial kernel; enet—elastic net; svmPoly—support vector machine (SVM) with a polynomial kernel; full—full feature set; lincomb—linear combinations filter; corr—high correlation filter; pca—principal component analysis.

Table S5. Predictive performance of the top 10 models in terms of mean cross-validated Brier Score and AUC for models on T1 CE sequence without edema mask.

Models Fit Only to CE Sequence without Edema Masks								
	Whole Tumor Mask				Necrotic and Enhancing Masks			
Rank	Model	Feature Selection	Brier Score Mean (95% CI)	Accuracy Mean (95% CI)	Model	Feature Selection	Brier Score Mean (95% CI)	Accuracy Mean (95% CI)
1	rf	corr	0.357 (0.262, 0.443)	0.752 (0.620, 0.843)	svmRad	pca	0.325 (0.255, 0.485)	0.782 (0.686, 0.860)
2	rf	lincomb	0.364 (0.276, 0.490)	0.759 (0.640, 0.863)	rf	corr	0.327 (0.261, 0.458)	0.791 (0.667, 0.880)
3	svmRad	corr	0.365 (0.244, 0.460)	0.749 (0.627, 0.843)	gbrm	full	0.329 (0.230, 0.473)	0.787 (0.725, 0.863)
4	rf	full	0.365 (0.273, 0.492)	0.756 (0.627, 0.882)	gbrm	lincomb	0.330 (0.219, 0.446)	0.775 (0.706, 0.843)
5	nnet	lincomb	0.367 (0.319, 0.442)	0.759 (0.660, 0.824)	svmRad	corr	0.331 (0.237, 0.425)	0.767 (0.680, 0.863)
6	svmRad	full	0.367 (0.258, 0.477)	0.757 (0.627, 0.882)	svmRad	full	0.342 (0.269, 0.443)	0.764 (0.667, 0.843)
7	nnet	pca	0.369 (0.239, 0.450)	0.750 (0.620, 0.863)	svmRad	lincomb	0.343 (0.263, 0.436)	0.764 (0.667, 0.843)
8	gbrm	lincomb	0.370 (0.296, 0.478)	0.761 (0.640, 0.843)	gbrm	corr	0.343 (0.237, 0.528)	0.777 (0.667, 0.863)
9	svmRad	pca	0.371 (0.266, 0.468)	0.750 (0.667, 0.863)	rf	full	0.346 (0.263, 0.498)	0.764 (0.627, 0.860)
10	svmPoly	lincomb	0.371 (0.233, 0.471)	0.745 (0.667, 0.863)	enet	full	0.351 (0.267, 0.454)	0.761 (0.667, 0.824)

rf—random forest; svmRad—SVM with a radial kernel; nnet—neural network; gbrm—generalized boosted regression model; svmPoly—support vector machine (SVM) with a polynomial kernel; Enet—elastic net; corr—high correlation filter; lincomb—linear combinations filter; full—full feature set; pca—principal component analysis.

Table S6. Feature importance for the highest performing models.

Using All (Multiparametric MRI) Sequences						
	GBRM Full—Whole Tumor (WT) and Edema Masks			GBRM Corr—Necrotic, Enhancing and Edema Masks		
Rank	Seq	Mask	Variable	Seq	Mask	Variable
1	T2	WT	firstorder_Energy	CE	Nec	ngtdm_Coarseness
2	CE	WT	firstorder_10Percentile	CE	Enh	ngtdm_Busyness
3	A	WT	gldm_DependenceNonUniformity	T2	Enh	ngtdm_Busyness
4	T1	WT	glszm_LargeAreaEmphasis	F	Enh	glcm_ClusterShade
5	A	WT	glszm_LargeAreaHighGrayLevelEmphasis	T2	Enh	shape_Sphericity
6	CE	WT	glrlm_RunLengthNonUniformity	T2	Nec	shape_Sphericity
7	T2	WT	ngtdm_Busyness	F	Nec	ngtdm_Coarseness
8	T1	WT	firstorder_Skewness	T1	Enh	glszm_SmallAreaLowGrayLevelEmphasis
9	F	WT	glcm_ClusterShade	T2	Enh	glcm_Imc1
10	T2	WT	glcm_JointAverage	T1	Enh	firstorder_Skewness
**Using Individual T1 CE Sequence**						
	**GBRM Full—Whole Tumor (WT) and Edema Masks**			**GBRM Full—Necrotic, Enhancing and Edema Masks**		
**Rank**	**Seq**	**Mask**	**Variable**	**Seq**	**Mask**	**Variable**
1	CE	WT	shape_Maximum2DDiameterRow	CE	Nec	ngtdm_Coarseness
2	CE	WT	shape_MeshVolume	CE	Enh	shape_MajorAxisLength
3	CE	WT	shape_Maximum2DDiameterColumn	CE	Enh	shape_Maximum2DDiameterRow
4	CE	WT	shape_Maximum2DDiameterSlice	CE	Enh	shape_SurfaceArea
5	CE	WT	firstorder_Skewness	CE	Enh	shape_Maximum2DDiameterSlice
6	CE	WT	ngtdm_Strength	CE	Enh	shape_Sphericity
7	CE	WT	firstorder_10Percentile	CE	Enh	ngtdm_Coarseness
8	CE	WT	glrlm_RunLengthNonUniformity	CE	Enh	shape_SurfaceVolumeRatio

9	CE	WT	shape_MajorAxisLength	CE	Enh	firstorder_Median
10	CE	WT	shape_Sphericity	CE	Enh	firstorder_Kurtosis
**Using T1 CE Sequence without Edema Mask**						
		**RF Corr—Whole Tumor (WT) Mask**			**RF Corr—Necrotic and Enhancing Masks**	
**Rank**	**Seq**	**Mask**	**Variable**	**Seq**	**Mask**	**Variable**
1	CE	WT	gldm_DependenceNonUniformity	CE	Enh	gldm_DependenceNonUniformity
2	CE	WT	ngtdm_Coarseness	CE	Nec	ngtdm_Coarseness
3	CE	WT	firstorder_10Percentile	CE	Enh	ngtdm_Coarseness
4	CE	WT	firstorder_Skewness	CE	Nec	shape_Sphericity
5	CE	WT	glszm_LargeAreaHighGrayLevelEmphasis	CE	Enh	shape_Sphericity
6	CE	WT	glszm_ZoneVariance	CE	Enh	firstorder_10Percentile
7	CE	WT	glcm_Correlation	CE	Enh	shape_Flatness
8	CE	WT	ngtdm_Busyness	CE	Enh	ngtdm_Busyness
9	CE	WT	ngtdm_Strength	CE	Enh	shape_Flatness
10	CE	WT	shape_Sphericity	CE	Enh	firstorder_Minimum

CE—T1 contrast enhanced; WT—whole tumor mask; Enh—enhancing mask; Nec—necrotic mask; gbrm—generalized boosted regression model; rf—random forest; corr—high correlation filter; full—full feature set.
